# Substantial Contribution of SmeDEF, SmeVWX, SmQnr, and Heat Shock Response to Fluoroquinolone Resistance in Clinical Isolates of *Stenotrophomonas maltophilia*

**DOI:** 10.3389/fmicb.2019.00822

**Published:** 2019-04-17

**Authors:** Chao-Jung Wu, Hsu-Feng Lu, Yi-Tsung Lin, Man-San Zhang, Li-Hua Li, Tsuey-Ching Yang

**Affiliations:** ^1^Department of Biotechnology and Laboratory Science in Medicine, National Yang-Ming University, Taipei, Taiwan; ^2^Department of Clinical Pathology, Cheng Hsin General Hospital, Taipei, Taiwan; ^3^Department of Restaurant, Hotel and Institutional Management, Fu-Jen Catholic University, New Taipei City, Taiwan; ^4^Division of Infectious Diseases, Department of Medicine, Taipei Veterans General Hospital, Taipei, Taiwan; ^5^School of Medicine, National Yang-Ming University, Taipei, Taiwan; ^6^Department of Pathology and Laboratory Medicine, Taipei Veterans General Hospital, Taipei, Taiwan; ^7^School of Medical Laboratory Science and Biotechnology, College of Medical Science and Technology, Taipei Medical University, Taipei, Taiwan

**Keywords:** *Stenotrophomonas maltophilia*, fluoroquinolone resistance, efflux pump, Qnr protein, heat shock response

## Abstract

*Stenotrophomonas maltophilia* is an emerging multi-drug resistant opportunistic pathogen. Although fluoroquinolones (FQ) are still clinically valuable for the treatment of *S. maltophilia* infection, an increasing prevalence in FQ resistance has been reported. Overexpression of SmeDEF, SmeVWX, and SmQnr, and de-repressed expression of heat shock response are reported mechanisms responsible for FQ resistance in *S. maltophilia*; nevertheless, some of these mechanisms are identified from laboratory-constructed mutants, and it remains unclear whether they occur in clinical setting. In this study, we aimed to assess whether these mechanisms contribute substantially to FQ resistance in clinical isolates. Eighteen ciprofloxacin- and levofloxacin-resistant isolates were selected from 125 clinical isolates of *S. maltophilia*. The expression of *smeE*, *smeW*, and *Smqnr* genes of these isolates was investigated by RT-qPCR. The de-repressed heat shock response was assessed by *rpoE* expression at 37°C and bacterial viability at 40°C. The contribution of SmeDEF, SmeVWX, and SmQnr, and heat shock response to FQ resistance was evaluated by mutants construction and susceptibility testing. The results demonstrated that simply assessing the overexpression of SmeDEF, SmeVWX, and SmQnr by RT-qPCR may overestimate their contribution to FQ resistance. Simultaneous overexpression of SmeDEF and SmeVWX did not increase the resistance level to their common substrates, but extended the resistance spectrum. Moreover, the de-repressed expression of heat shock response was not observed to contribute to FQ resistance in the clinical isolates of *S. maltophilia*.

## Introduction

Fluoroquinolone (FQ) acts as a bactericidal antibiotic by inhibiting the activity of type II topoisomerase, DNA gyrase and topoisomerase IV, which are necessary for the replication of DNA. Owing to its good potency, wide spectrum of activity, and oral bioavailability, FQ is used extensively for clinical indications. FQ resistance is emerging with clinical use and becoming common in some bacterial pathogens. The reported FQ resistance mechanisms in gram-negative bacteria include mutation or downexpression of quinolone-entrance porins, mutations in DNA gyrase and/or topoisomerase IV, and overexpression of aminoglycoside modifying enzyme, quinolone-extrusion pumps or Qnr proteins (Aldred et al., 2014; Hooper and Jacoby, 2015).

In addition to the traditionally known resistance mechanisms, bacterial stress responses have also been considered as determinants of antibiotic resistance (Poole, 2012). During bacterial growth, bacteria encounter a variety of stresses and elicit specific stress responses for their survival. These stress responses not only protect bacteria from the encountered stress, but also promote physiological changes within them that can indirectly compromise or enhance antibiotic resistance (Poole, 2012). The heat shock response has been linked to FQ resistance in *Escherichia coli* (Yamaguchi et al., 2003) and in *Stenotrophomonas maltophilia* (Bernardini et al., 2015).

*Stenotrophomonas maltophilia* is ubiquitous in the environment and can be isolated from water, soil, plants, and humans (Brooke, 2012). Therefore, *S. maltophilia* has equipped itself with many stress response capabilities to face variable environmental challenges. In addition, *S. maltophilia* is an opportunistic pathogen that is responsible for many nosocomial infections. Treatment of *S. maltophilia* infections is challenging because this bacterium is intrinsically resistant to many antibiotics including β-lactams, aminoglycosides, and macrolides (Sánchez, 2015). Trimethoprim/sulfamethoxazole, ceftazidime, ticarcillin/clavulanate, minocycline, doxycycline, tigecycline, and FQ are the available antimicrobial drugs of choice (Looney et al., 2009). Although FQ shows *in vivo* activity against *S. maltophilia*, FQ-resistant isolates have been increasingly reported in recent years (Chong et al., 2017). FQ resistance associated with mutation or downexpression of porins has been documented in other gram-negative bacteria (Doménech-Sánchez et al., 2003; Hooper and Jacoby, 2015), but not in *S. maltophilia*. Despite some mutations in genes encoding topoisomerases of *S. maltophilia* being reported, the linkage between these mutations and FQ resistance has not been fully supported (Ribera et al., 2002; Valdezate et al., 2002). The best studied mechanisms of FQ resistance in *S. maltophilia* are the overexpression of multidrug efflux pumps SmeDEF and SmeVWX, and chromosomally encoded Qnr protein (SmQnr) (Alonso and Martinez, 2000; Chang et al., 2011; Chen et al., 2011). In addition to the known mechanisms of FQ resistance, a stress-response dependent resistance mechanism has been proposed recently. Bernardini et al. (2015) proposed a close linkage between de-repressed expression of heat shock response and reduced FQ susceptibility. Nevertheless, in some cases, the mechanisms were identified in laboratory-constructed mutants, and it remains unclear whether the resistance mechanisms are found in clinical FQ-resistant isolates and if they make a substantial contribution to FQ resistance. This theme is discussed in this article.

## Materials and Methods

### Bacterial Strains, Plasmids, Primers, and Culture Conditions

The strains and plasmids used in this study are listed in Supplementary Table S1. Supplementary Table S2 is the PCR primers list. All primers used in this study were designed based on the genome of *S. maltophilia* K279a (Crossman et al., 2008). The bacteria were cultured aerobically at 37°C with agitation in Lysogeny-Broth (LB) broth. The bacterial growth was monitored by measuring the optical density of bacterial culture at 450 nm instead of at 600 nm, because 450 nm is a more sensitive wavelength to reflect the growth of *S. maltophilia* than 600 nm. An A_450__nm_ of 1 corresponds to 3.6 × 10^8^ cells/ml (Hu et al., 2008).

### Antimicrobial Susceptibility Test

The antibiotics susceptibilities of the bacteria were tested by twofold agar dilution method according to the guideline of Clinical Laboratory Standards Institute (CLSI) (CLSI, 2017). The minimal inhibitory concentration (MIC) was recorded as the lowest concentration of the antibiotic that completely inhibited bacterial growth. The MICs were determined in triplicate by serial dilutions in Muller-Hinton (MH) agar. Antibiotics (ciprofloxacin, levofloxacin, chloramphenicol, tetracycline, erythromycin, and leucomycin) were purchased from Sigma Chemical Co.

### RNA Preparation and Quantitative Real-Time PCR (RT-qPCR)

The DNA-free RNA of logarithmic-phase *S. maltophilia* cells were extracted using Total RNA Extraction Kit Mini (ARROWTEC, New Taipei City, Taiwan). RNA quantity and quality were assessed by absorption spectrometry and gel electrophoresis, respectively. RNA (400 ng) was reverse transcribed to cDNA by High Capacity cDNA Reverse Transcription Kit (Applied Biosystems, Foster City, CA, United States) and random hexamer primers. RT-qPCR was performed on 10 ng of cDNA per reaction volume by the ABI Prism 7000 Sequence Detection System (Applied Biosystems, Foster City, CA, United States) using the Smart Quant Green Master Mix (Protech Technology Enterprise Co., Ltd., New Taipei City, Taiwan) according to the manufacturer’s protocols. The amplification program consisted of one holding stage at 95°C for 20 s, followed by 40 cycling stages at 95°C for 12 s and 60°C for 30 s. The transcripts were normalized with the internal control 16s rRNA gene using ΔΔC_T_ method (Livak and Schmittgen, 2001), where C_T_ is the threshold cycle. Primers used for RT-qPCR were listed in Supplementary Table S2. All experiments were performed in triplicate.

### High Temperature Cell Viability Assay

Overnight-cultured bacteria cells were inoculated in to fresh LB with an initial OD_450__nm_ of 0.15 and then further cultured for 5 h. The exponential-phased bacteria cells were collected, adjusted to 2 × 10^8^ CFU/ml, and 10-fold serially diluted in LB broth. Then five microliter of the bacterial cells were spotted on LB agars and incubated at either 37 or 40°C. After 18 h, the colony growth was observed.

### Construction of Deletion Mutants

The deletion mutants were constructed by the strategy of double-crossover homologous recombination. Recombinant plasmids pΔDEF (Wu et al., 2016), pΔ5 (Chen et al., 2011), pΔQnr (Chang et al., 2011), and pΔRpoE (Huang et al., 2014) were used for the construction of *smeDEF*, *smeU1VWU2X*, *Smqnr*, and *rpoE* mutants, respectively. The plasmid mobilization, transconjugant selection, and mutant confirmation were performed as described previously (Yang et al., 2009). Considering the usage of tetracycline as the selection pressure during deletion mutant construction, clinical isolates of low tetracycline MIC values were given priority for deletion mutant construction.

### Construction of pRpoH and pSmqnrC

The 1610-bp PCR amplicon, containing an intact *rpoH* gene, was amplified using primer set of RpoH-F/RpoH-R, digested with *Hin*dIII and *Sac*I enzymes, and then cloned into expression plasmid pRK415 (Keen et al., 1988) to yield pRpoH (Supplementary Table S1). The recombinant plasmid was transformed into wild-type KJ to obtain KJ(pRpoH). Similar strategy was used for the construction of pSmqnrC by cloning 2186-bp *SmqnrR*-*Smqnr* cluster DNA [PCR by SmqnrC-F and SmqnrC-R primers (Supplementary Table S2)] into pRK415, generating pSmqnrC (Supplementary Table S1).

### Ethics Statement

This study was carried out in accordance with the recommendations of “Gene Recombinant Experiment Applications” and “Biological Materials Applications,” Biosafety Committee, National Yang-Ming University. The protocol was approved by the Biosafety Committee. All subjects gave written informed consent in accordance with the Declaration of Helsinki.

## Results

### FQ Susceptibility of Clinical Isolates

To investigate the underlying FQ resistance mechanisms of *S. maltophilia*, 125 clinical isolates were selected from different sources (sputum, 75; aspirate of respiratory tract, 19; blood, 9; urine, 6; pus/wound, 4; ascites, 4; drainage, 4; abscess, 2; and pleural fluid, 2) and their MICs for FQ were determined by the agar dilution method. The antimicrobials tested included ciprofloxacin and levofloxacin. According to CLSI guidelines, FQ resistance is defined as the MIC values for levofloxacin (LEV) ≥8 μg/ml for *S. maltophilia*, and ciprofloxacin (CIP) ≥4 μg/ml for *Pseudomonas aeruginosa* (no available reference for *S. maltophilia*). Among a total of 125 clinical isolates, 18 isolates were resistant to FQ. Herein, we collected the 18 isolates (Table 1) to elucidate the underlying resistance mechanisms with respect to overexpression of *smeDEF*, *smeVWX*, and *Smqnr*, and heat shock response-mediated protection.

**Table 1:** Antibiotic susceptibility and the expressions of *smeE*, *smeW*, *smqnr*, and *rpoE* genes of *S. maltophilia* isolates.

	MIC^a^ (μg/ml)						RT-qPCR^b^ (fold)			
	FQ		CHL	TET	Macrolide		*smeE*	*smeW*	*smqnr*	*rpoE*
Isolate	CIP	LEV			ERV	LM				
KJ	1	1	8	16	64	256	1	1	1	1/5.9 ± 1.9^c^
KJΔRseA	1	1	8	16	64	256	0.9 ± 0.1	1.3 ± 0.2	1.5 ± 0.1	4.5 ± 2.3
KJΔRpoEΔRseA	1	1	8	16	64	256	1.3 ± 0.3	0.8 ± 0.1	0.7 ± 0.2	– –
V47	16	16	128	16	128	1024	4.9 ± 0.1	6.5 ± 1.0	5.7 ± 0.0	5.5 ± 1.3
V53	8	16	32	16	512	4096	69.0 ± 4.0	0.5 ± 0.4	2.6 ± 0.4	2.4 ± 0.5
V56	16	8	64	128	512	2048	0.7 ± 0.0	18.4 ± 1.4	1.9 ± 0.1	14 ± 9.5
V61	8	8	32	64	512	4096	48.4 ± 2.7	0.9 ± 0.0	15.7 ± 4.5	5.7 ± 0.4
V62	8	8	8	128	128	4096	0.7 ± 0.0	0.3 ± 0.0	0.8 ± 0.0	10.5 ± 1.5
V63	8	8	32	64	512	4096	67.4 ± 14.1	0.6 ± 0.0	2.1 ± 0.1	2.7 ± 0.2
V78	32	32	64	256	256	2048	54.6 ± 8.4	0.6 ± 0.1	58.9 ± 6.8	8.5 ± 1.7
V80	8	16	32	64	256	4096	0.6 ± 0.0	0.5 ± 0.2	0.7 ± 0.0	3.9 ± 0.7
V82	4	8	128	16	256	2048	21.4 ± 0.8	0.6 ± 0.0	1.3 ± 0.1	0.4 ± 0.1
V84	16	16	32	64	512	4096	99.7 ± 18.6	0.2 ± 0.0	1.8 ± 0.0	12.9 ± 2.0
V90	8	16	16	32	512	4096	73.7 ± 6.4	38.0 ± 0.5	4.9 ± 3.1	2.1 ± 1.6
V93	16	32	32	64	512	4096	92.3 ± 4.1	0.6 ± 0.0	2.3 ± 0.1	0.5 ± 0.1
V95	8	16	64	16	256	2048	3.2 ± 0.0	4.4 ± 1.6	0.3 ± 0.1	1.5 ± 0.5
V96	32	32	64	256	256	1024	6.1 ± 0.4	0.6 ± 0.2	4.9 ± 0.6	2.8 ± 0.2
V99	8	16	128	8	256	1024	0.5 ± 0.0	8.6 ± 0.8	0.4 ± 0.0	5.4 ± 0.9
V101	4	8	64	16	128	1024	4.3 ± 1.6	0.5 ± 0.1	20.2 ± 2.1	2.9 ± 1.4
V104	8	16	32	64	256	4096	59.3 ± 3.3	7.8 ± 1.6	4.9 ± 0.0	2.1 ± 1.1
V105	8	16	32	64	256	4096	0.4 ± 0.0	0.7 ± 0.0	0.5 ± 0.0	3.3 ± 0.3


*^*a*^MIC, minimal inhibitory concentration. ^*b*^The transcript levels of *smeE*, *smeW*, *Smqnr*, and *rpoE* in the clinical isolates were normalized with those in *S. maltophilia* KJ, a quinolone susceptible strain, cultured at 37°C. All isolates were cultured at 37°C, except KJ cells, which were cultured at 37 and 42°C. ^c^The relative *rpoE* transcript of KJ cells at 37 and 42°C. FQ, fluoroquinolone; CIP, ciprofloxacin; LEV, levofloxacin; ERY, erythromycin; LM, leucomycin; CHL, chloramphenicol; TET, tetracycline.*

### Association of the Overexpression of the SmeDEF and SmeVWX Pumps With FQ Resistance

Considering the susceptibility of an isolate to other antibiotics, in addition to FQ, is important for elucidating the involvement of efflux pumps in FQ resistance. MICs of erythromycin, leucomycin, chloramphenicol, and tetracycline were tested for the 18 FQ-resistant isolates and the results are summarized in Table 1. The antibiotic susceptibility of isolate KJ, a FQ-susceptible strain whose characteristics have been reported previously (Chen et al., 2011), was used as a control. Table 1 demonstrates that most of the FQ-resistant isolates were also resistant to other antibiotics, exhibiting the multidrug-resistant phenotype, and highly suggests the involvement of overexpression of multidrug efflux pumps.

To assess the possible contribution of overexpression of SmeDEF and SmeVWX pumps to FQ resistance, the *smeE*, and *smeW* transcripts in the 18 FQ-resistant isolates were validated by RT-qPCR. Compared to that in KJ, the transcript in the isolates assayed had to have at least a threefold increment in levels to be considered as being overexpressed. Of the 18 FQ-resistant isolates, 13 (72%) isolates overexpressed *smeE*, six (33%) isolates overexpressed *smeW*, and four (22%) isolates simultaneously overexpressed *smeE* and *smeW* (Table 1). To test whether the SmeDEF and SmeVWX overexpression indeed contributes to FQ resistance, the strategy of genetic knockout was performed. Since the selection of pEX18Tc-mediated transconjugants was based on the tetracycline resistance (Hoang et al., 1998), the clinical isolates of low tetracycline MIC values were prone to deletion mutants construction. Hence, isolates V53, V99, and V47 were selected. The *smeDEF* and *smeU1VWU2X* operons were deleted from the chromosomes of isolates V53, V99, and V47, separately or together, to yield V53ΔDEF (a *smeDEF* deletion mutant of isolate V53), V99Δ5 (a *smeU1VWU2X* deletion mutant of isolate V99), V47ΔDEF (a *smeDEF* deletion mutant of isolate V47), V47Δ5 (a *smeU1VWU2X* deletion mutant of isolate V47), and V47ΔDEFΔ5 (a *smeDEF* and *smeU1VWU2X* deletion double mutant of isolate V47). Compared to its parental strain, V99Δ5 exhibited decreased resistance to FQ and chloramphenicol (Table 2), consistent with the substrate profiles of SmeVWX (Chen et al., 2011), indicating that overexpression of SmeVWX pumps contributes to FQ resistance in isolate V99. Compared to the parental isolate V47, V47Δ5 showed decreased resistance to chloramphenicol and FQ, and V47ΔDEF showed increased susceptibility to tetracycline and macrolide. An unexpected result was observed in isolate V53; inactivation of SmeDEF did not affect its susceptibility to FQ tested (Table 2). Because of this inconsistency, another two SmeDEF-overexpression isolates, V63 and V82, were chosen for further investigation. As in isolate V53, deletion of *smeDEF* in isolate V82 had no impact on its susceptibility to the antibiotics tested. However, deletion of *smeDEF* decreased the MICs of FQ, macrolide, chloramphenicol, and tetracycline, the known substrates of SmeDEF pump, for isolate V63 (Table 2; Alonso and Martinez, 2000). To check whether the *smeE* transcript overexpression in isolates V53 and V82 (Table 1) is caused by the primers bias, we sequenced the RT-qPCR products and found that they were exactly *smeE*.

**Table 2:** Antibiotic susceptibilities of clinical isolates and their derived mutants.

Strain	MIC^a^ (μg/ml)					
	FQ				Macrolide	
	CIP	LEV	CHL	TET	ERY	LM
KJ	1	1	8	16	64	256
KJΔDEF	0.5	0.5	4	8	32	128
KJΔ5	1	1	8	16	64	256
KJΔQnr	1	1	8	32	64	256
KJΔRseA	1	1	8	16	64	256
KJΔRpoE	1	1	8	16	64	256
KJΔRpoEΔRseA	1	1	8	16	64	256
V53	8	16	32	16	512	4096
V53ΔDEF	8	16	32	16	512	4096
V63	8	8	32	64	512	4096
V63ΔDEF	0.5	0.125	4	4	16	64
V82	4	8	128	16	256	2048
V82ΔDEF	4	8	128	16	256	2048
V99	8	16	128	8	256	1024
V99Δ5	0.5	1	16	8	256	1024
V47	16	16	128	16	128	1024
V47ΔDEF	16	16	128	8	32	256
V47Δ5	0.25	0.5	8	16	128	1024
V47ΔDEFΔ5	0.25	0.5	8	8	32	128
V47ΔSmqnr	4	4	128	16	128	512
V47ΔSmqnr(pSmqnrC)	8	8	128	16	128	512
V47ΔRpoE	16	16	128	16	128	1024
V61	8	8	32	64	512	4096
V61ΔRpoE	8	8	32	64	512	4096
V84	16	16	32	64	512	4096
V84ΔRpoE	16	16	32	64	512	4096


*^*a*^MIC, minimal inhibitory concentration. FQ, fluoroquinolone; CIP, ciprofloxacin; LEV, levofloxacin; ERY, erythromycin; LM, leucomycin; CHL, chloramphenicol; TET, tetracycline.*

### Association of Smqnr Overexpression With FQ Resistance

A *Smqnr* gene carried by chromosomes is known to contribute to low-level FQ resistance in *S. maltophilia* (Sanchez and Martinez, 2010), but it is not well conserved in all clinical isolates (Jia et al., 2015). Hence, the prevalence of the *Smqnr* gene was first investigated by PCR, and was found to be 100% in the 18 FQ-resistant isolates. The expression levels of the *Smqnr* gene in the 18 *Smqnr*-harboring isolates were further assessed by RT-qPCR, and seven (39%) isolates displayed abundant levels of the *Smqnr* transcript compared to KJ (Table 1). Isolate V47 was selected for *Smqnr* mutant construction and confirmation of the contribution of SmQnr to FQ resistance. As expected, inactivation of *Smqnr* of isolate V47 increased the susceptibility to FQ and the resistance was partially reverted by complementation of pSmqnrC (Table 2).

### Association of Heat Shock Response With FQ Resistance

In general, the heat shock response is triggered by specific sigma factors, *σ*^E^ and(or) *σ*^32^, in many gram-negative bacteria (Rouvière et al., 1995); nevertheless the sigma factor(s) responsible for the heat shock response in *S. maltophilia* is still unrevealed. Hence, the expression levels of the *rpoE* and *rpoH* genes of *S. maltophilia* were firstly determined with or without heat shock by RT-qPCR. After treatment at 42°C for 10 min, the *rpoE* expression showed a 5.9 ± 1.9-fold increment (Table 1), whereas *rpoH* expression level was not significantly changed in logarithmic-phase KJ (a FQ-susceptible strain). Next, *rpoE* and *rpoH* in-frame deletion mutants and overexpression constructs were prepared for investigation of their involvement in the heat shock response. KJΔRseA and KJΔRpoEΔRseA are representatives of *σ*^E^ overexpression and *rpoE* deletion, respectively (Huang et al., 2014). RpoH overexpression construct, KJ(pRpoH), was available; however, after several attempts, we could not successfully obtain the *rpoH* mutant. It seems that *σ*^32^ is required for viability of *S. maltophilia*.

**FIGURE 1:**
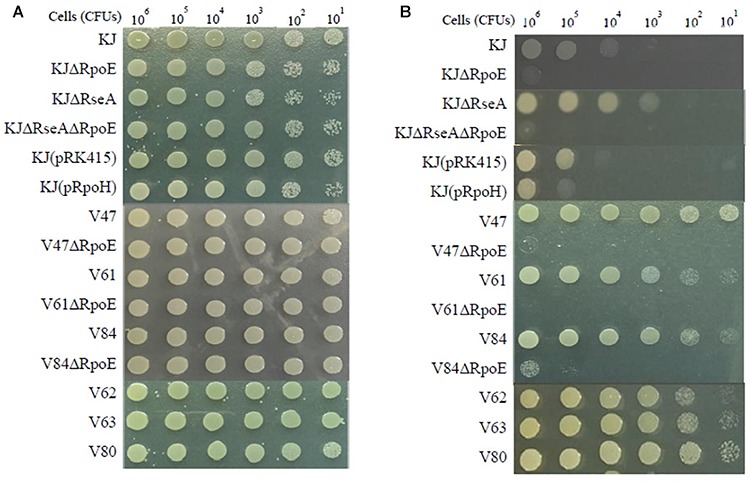
Viability of *S. maltophilia* isolates and their derived mutants after heat shock. The logarithmic-phase bacterial cells were collected, adjusted to 2 × 10^5^ CFUs/μl, and 10-fold serially diluted. Then 5 μl of the bacterial cells were spotted on LB agar. After an 18-h incubation period, bacterial cell growth was observed. **(A)** Incubation at 37°C. **(B)** Incubation at 40°C.

If a bacterium is in a situation of de-repression of heat shock response, it has a viability superiority at high temperature. The high-temperature cell viability of the wild-type KJ and its derived mutants was assessed. Since KJ grew poorly at 42°C (data not shown), we tested the bacterial viability of the cells at 40°C. Compared to that of KJΔRseA, the viability at 40°C of KJΔRpoEΔRseA was severely compromised, indicating that RpoE is the key sigma factor for heat shock response. This is supported by similar results comparing the viabilities of KJ and KJΔRpoE at 40°C (Figure 1B). Nevertheless, KJ(pRpoH) did not display better viability at 40°C than KJ(pRK415) (Figure 1B).

To decipher whether de-repressed expression of the heat shock response occurs in clinical FQ-resistant isolates, two approaches were implemented to assess whether an isolate had de-repressed expression of heat shock response: *rpoE* expression level at 37°C and bacterial viability at 40°C. *rpoE* expression of the 18 FQ-resistant isolates at 37°C was determined by RT-qPCR. Meanwhile, the *rpoE* expression of KJ at 37 and 42°C was measured as the control. The levels of *rpoE* transcripts of seven isolates (V47, V56, V61, V62, V78, V84, and V99) were higher than that of KJ at 37°C and almost equal or even higher than that of KJ at 42°C (Table 1). On the other hand, we considered the cell viability at high temperature. Of the 18 FQ-resistant isolates assayed, six isolates (V47, V61, V62, V63, V80, and V84) exhibited better viability at 40°C than strains KJ and KJΔRseA (Figure 1). The isolates with an abundant *rpoE* expression at 37°C and superior viability at 40°C were considered as having de-repressed expression of heat shock response. Of the 18 FQ-resistant isolates, four (V47, V61, V62, and V84) met the criteria.

To clarify the relationship between heat shock response and FQ resistance, *rpoE* deletion mutants of the four isolates V47, V61, V62, and V84 were individually constructed. After several tries, the *rpoE* deletion mutant of V62 was not available, highly because of its tetracycline MIC as high as 128 μg/ml. The 40°C viability and FQ susceptibility of V47ΔRpoE, V61ΔRpoE, and V84ΔRpoE were investigated. At the same time, the FQ susceptibilities of KJΔRseA and KJΔRpoEΔRseA were also determined to evaluate the impact of *rpoE*-dependent heat shock response on the FQ susceptibility in a FQ-susceptible strain KJ. As expected, inactivation of *rpoE* compromised the viability at 40°C for strains KJΔRseA, V47, V61, and V84 (Figure 1B). However, KJΔRseA, KJΔRpoEΔRseA, V47ΔRpoE, V61ΔRpoE, and V84ΔRpoE displayed comparable FQ susceptibilities to their own parental strains (Table 2).

## Discussion

In considering efflux pump-mediated antibiotic resistance, it is plausibly accepted that increased expression of RND pumps might increase resistance to a variety of antibiotics that are substrates of the efflux pump (Garcia-Leon et al., 2015). RT-qPCR is the general strategy used in previous studies for investigating the correlation between resistance genes overexpression and FQ resistance in clinical isolates. Distinct from previous studies, we tried to elucidate the substantial contribution of resistance genes overexpression to FQ resistance in clinical isolates by the strategy of deletion mutants construction. Isolates V53 and V82 in this study provided examples that *smeDEF* overexpression, assessed by qRT-PCR, did not substantially contribute to resistance. This observation supports that past studies may have overestimated the contribution of efflux pumps to antibiotic resistance and omitted other ancillary resistance mechanisms. The inconsistency between *smeDEF* expression and the susceptibility phenotypes of isolates V53 and V82 could be due to the loss-of-function mutation in *smeDEF* genes, improper assembly of in SmeDEF, and(or) other unidentified resistance mechanisms. Absolutely, we cannot deny the possibility that deletion mutants present additional unidentified mutations conferring the observed phenotypes at this moment.

The substrate profiles of SmeDEF and SmeVWX pumps partially overlap, such as those for chloramphenicol, tetracycline, and quinolone (Alonso and Martinez, 2000; Chen et al., 2011). Some interesting findings were observed from isolate V47: (i) simultaneous overexpression of SmeDEF and SmeVWX in V47 appears not to increase the resistance level to their common substrates (Table 2), but extends the resistance spectrum; (ii) compared to parental strain V47, V47Δ5 displays comparable tetracycline susceptibility and V47ΔDEF has comparable susceptibility to chloramphenicol and quinolone, supporting the view that SmeDEF is a more potent pump than SmeVWX in the tetracycline extrusion, and SmeVWX has a higher efficiency for extrusion of chloramphenicol and quinolone than SmeDEF.

Two sigma factors, *σ*^E^ and *σ*^32^, are known to be involved in the heat shock response in many gram-negative bacteria including *E. coli*, *P. aeruginosa*, and *Burkholderia pseudomallei* (Rouvière et al., 1995; Potvin et al., 2008; Vanaporn et al., 2008). In this study, we demonstrated that *σ*^E^ plays a critical role in the heat shock response of *S. maltophilia*; however, the role of *σ*^32^ in the heat shock response is still unclear because we could not obtain *rpoH* mutants. The unavailability of *rpoH* mutants implies the importance of *σ*^32^ in *S. maltophilia*. The fact that *σ*^32^ is important for viability has been described for *Neisseria gonorrhoeae* and *Francisella tularensis* (Du et al., 2005; Grall et al., 2009). A more interesting finding in this study is that there are some isolates that overexpressed *rpoE* but with no better viability at 40°C, such as isolates V56, V78, and V99. Two possible explanations are proposed herein. (i) The σ^E^ of these isolates may have a compromised function and thus cannot trigger an adequate heat shock response. (ii) Some unidentified mechanisms occur in these isolate, which antagonize σ^E^-mediated heat shock response.

The interplay among the heat shock response, *σ*^E^, and FQ resistance has been reported. *E. coli* exposed to levofloxacin induce the synthesis of heat shock proteins, such as DnaK, GrpE, GroEL, GroES, LbpA, and LbpB (Yamaguchi et al., 2003). Disruption of some heat shock genes, such as *ΔrpoH*, *ΔdnaK*, *ΔgroEL*, and *Δlon*, increase the susceptibility of *E. coli* to levofloxacin (Yamaguchi et al., 2003). Inactivation of *rpoE* in *S. enterica* and *C. glutamicum* results in the elevation and reduction of quinolone resistance, respectively (Park et al., 2008; Xie et al., 2016). Recently, Bernardini et al. (2015) have reported that heat shock response activation and quinolone resistance elevation are simultaneously observed in an RNase G mutant of *S. maltophilia*. If the heat shock response indeed contributes to FQ resistance, KJΔRseA, a strain of de-repression expression of heat shock response, should have a FQ resistance phenotype; nevertheless, KJ, KJΔRseA, and KJΔRpoEΔRseA have comparable FQ susceptibility in our assayed system (Table 2). In addition, based on the results presented in this article, heat shock response and *σ*^E^ did not contribute substantially to FQ resistance in clinical setting; however, our findings do not totally deny the possible contribution of the heat shock response or *σ*^E^ to bacterial biological tolerance to quinolone.

## Author Contributions

C-JW, H-FL, M-SZ, and L-HL performed the experiments. Y-TL and T-CY designed and interpreted the results. All authors reviewed and revised the article, and approved it for publication.

## Conflict of Interest Statement

The authors declare that the research was conducted in the absence of any commercial or financial relationships that could be construed as a potential conflict of interest.
