# Supplementary material for: Substantial Contribution of SmeDEF, SmeVWX, SmQnr, and Heat Shock Response to Fluoroquinolone Resistance in Clinical Isolates of Stenotrophomonas maltophilia
Source: Front Microbiol. 2019 Apr 17;10:822. doi: 10.3389/fmicb.2019.00822 (PMC6479208; doi:10.3389/fmicb.2019.00822)
Supplement: Supplementary file 1 [file Table_1.pdf]

Table S1 Bacterial strains and plasmids used in this study

Strain or plasmid	Genotype or properties	Reference
S. maltophilia		
KJ	A clinical quinolone-susceptible isolate	Hu et al. 2008
KJΔDEF	*S. maltophilia* KJ mutant of *smeDEF* operon; Δ*smeDEF*	Huang et al. 2017
KJΔ5	*S. maltophilia* KJ mutant of *smeU1VWU2X* operon; Δ*smeU1VWU2X*	Chen et al. 2011
KJΔQnr	*S. maltophilia* KJ mutant of *Smqnr*; Δ*Smqnr*	Chang et al. 2011
KJΔRpoE	*S. maltophilia* KJ mutant of *rpoE*; Δ*rpoE*	Huang et al. 2014
KJΔRseA	*S. maltophilia* KJ mutant of *rseA*; Δ*rseA*	Huang et al. 2014
KJΔRpoEΔRseA	*S. maltophilia* KJ mutant of *rseA* and *rpoE*; Δ*rseA*, Δ*rpoE*	Huang et al. 2014
V53	A clinical quinolone-resistant isolate	This study
V53ΔDEF	*S. maltophilia* V53 mutant of *smeDEF* operon; Δ*smeDEF*	This study
V63	A clinical quinolone-resistant isolate	This study
V63ΔDEF	*S. maltophilia* V63 mutant of *smeDEF* operon; Δ*smeDEF*	This study
V82	A clinical quinolone-resistant isolate	This study
V82ΔDEF	*S. maltophilia* V82 mutant of *smeDEF* operon; Δ*smeDEF*	This study
V99	A clinical quinolone-resistant isolate	This study
V99Δ5	*S. maltophilia* V99 mutant of *smeU1VWU2X* operon; Δ*smeU1VWU2X*	This study
V47	A clinical quinolone-resistant isolate	This study
V47ΔDEF	*S. maltophilia* V47 mutant of *smeDEF* operon; Δ*smeDEF*	This study
V47Δ5	*S. maltophilia* V47 mutant of *smeU1VWU2X* operon; Δ*smeU1VWU2X*	This study
V47ΔDEFΔ5	*S. maltophilia* V47 mutant of *smeDEF* and *smeU1VWU2X* operons; *ΔsmeDEF*, Δ*smeU1VWU2X*	This study
V47ΔSmqnr	*S. maltophilia* V47 mutant of *Smqnr*;	This study
	Δ*Smqnr*	This study
V47ΔRpoE	*S. maltophilia* V47 mutant of *rpoE*; Δ*rpoE*	This study
V84	A clinical quinolone-resistant isolate	This study
V84ΔRpoE	*S. maltophilia* V84 mutant of *rpoE*; Δ*rpoE*	This study
V61	A clinical quinolone-resistant isolate	This study
V61ΔRpoE	*S. maltophilia* V61 mutant of *rpoE*; Δ*rpoE*	
E. coli		
DH5a	F- φ80d/*acZΔM15* Δ(*lacZYA-argF*)*U169 deoR recA1 endA1 hsdR17* (r_k^- m_k^+) *phoA supE44λ*	Invitrogen

S17-1	*thi-1 gyrA96 relA1* λ pir+ mating strain	Simon et al. 1986
**Plasmids**		
pEX18Tc	*sacB oriT*, Tcr	Hoang et al. 1998
pRK415	Mobilizable broad-host-range plasmid cloning vector, RK2 origin; Tcr	Keen et al. 1988
pΔDEF	pEX18Tc with an internal-deletion *smeDEF* operon; Tcr	Huang et al. 2017
pΔ5	pEX18Tc with an internal-deletion *smeU1VWU2X* operon; Tcr	Chen et al. 2011
pΔQnr	pEX18Tc with an internal-deletion *smqnr* gene; Tcr	Chang et al. 2011
pΔRpoE	pEX18Tc with an internal-deletion *rpoE* gene; Tcr	Huang et al. 2014
pΔRseA	pEX18Tc with an internal-deletion *rseA* gene; Tcr	Huang et al. 2014
pRpoH	pRK415 with an intact *rpoH* gene; Tcr	This study
pSmqnrC	pRK415 with an intact *SmqnrR-Smqnr* cluster; Tcr	This study

Chang, Y. C., Tsai, M. J., Huang, Y. W., Chung, T. C., and Yang, T. C. (2011). SmeQnrR, a DeoR-type transcriptional regulator, negatively regulates the expression of Sm*qnr* and Sm*tcrA* in *Stenotrophomonas maltophilia*. *J. Antimicrob. Chemother.* 66, 1024-1028.

Chen, C. H., Huang, C. C., Chung, T. C., Hu, R. M., Huang, Y. W., and Yang, T. C. (2011). Contribution of resistance-nodulation-division efflux pump operon *smeU1-V-W-U2-X* to multidrug resistance of *Stenotrophomonas maltophilia*. *Antimicrob. Agents Chemother.* 55, 5826-5833.

Hoang, T. T., Karkhoff-Schweizer, R. R., Kutchma, A. J., and Schweizer, H. P. (1998). A broad-host-range Flp-FRT recombination system for site-specific excision of chromosomally-located DNA sequences: application for isolation of unmarked *Pseudomonas aeruginosa* mutants. *Gene.* 212, 77-86.

Hu, R. M., Huang, K. J., Wu L. T., Hsiao, Y. J., and Yang, T. C. (2008). Inductin of L1 and L2 β-lactamases of *Stenotrophomonas maltophilia*. *Antimicrob. Agents Chemother.* 52,1198-1200.

Huang, Y. W., Lin, C. W., Ning, H. C., Lin, Y. T., Chang, Y. C., and Yang, T. C. (2017). Overexpression of SmeDEF efflux pump decrease aminoglycoside resistance in *Stenotrophomonas maltophilia*. *Antimicrob. Agents Chemother.* 61, e02685-16.

Huang, Y. W., Liou, R. S., Lin, Y. T., Huang, H. H., and Yang, T. C. (2014). A linkage between

SmeIJK efflux pump, cell envelope integrity, and σ^E-mediated envelope stress response in *Stenotrophomonas maltophilia*. *PLoS One* 9, e111784.

Keen, N. T., Tamaki, S., Kobayashi, D., and Trollinger, D. (1988). Improved broad-host-range plasmids for DNA cloning in gram-negative bacteria. *Gene*. 70, 191-197.

Simon, R., O'Connell, M., Labes, M., and Puhler, A. (1986). Plasmid vectors for the genetic analysis and manipulation of rhizobia and other gram-negative bacteria. *Methods Enzymol*. 118, 640-659.
