# Supplementary material for: Substantial Contribution of SmeDEF, SmeVWX, SmQnr, and Heat Shock Response to Fluoroquinolone Resistance in Clinical Isolates of Stenotrophomonas maltophilia
Source: Front Microbiol. 2019 Apr 17;10:822. doi: 10.3389/fmicb.2019.00822 (PMC6479208; doi:10.3389/fmicb.2019.00822)
Supplement: Supplementary file 2 [file Table_2.pdf]

Table S2 PCR primers used in this study

Primer	Sequences	Amplicon (bp)	Reference
SmqnrC-F	5'-GCAAGCTTCTGGCCACCATAA-3'	2186	This study
SmqnrC-R	5'-GCTCTAGAACCCTGCACTTC-3'		This study
RpoH-F	5'- GTTCAAGCTTTGGGGTGCCTA 3'	1610	This study
RpoH-R	5'- CACGAGCTCCTATCGGTCAGTGA-3'		This study
SmeEQ-F	5'- GCAGCTCAACGCCACCATCAAC -3'	250	Chen et al. 2011
SmeEQ-R	5'- TTCCAGCGCGGCATTCACAC -3'		Chen et al. 2011
SmeWQ-F	5'- GCCCACACCATCTCGTTCCC -3'	221	Chen et al. 2011
SmeWQ-R	5'- TAGCCGTTGCCGTTGCCC -3'		Chen et al. 2011
SmqnrQ-F	5'- TTCTACGATGCCGACAGCC -3'	279	This study
SmqnrQ-R	5'- CCACAGCTCGACTTTTCC-3'		This study
RpoEQ-F	5'- CGCCGACCGATGACATCGAC -3'	254	Chen et al. 2011
RpoEQ-R	5'- CGCCCGGAAGATCCGTGAAC -3'		Chen et al. 2011
RpoHQ-F	5'- GCCGACCGACGAGGACAAC 3'	263	This study
RpoHQ-R	5'- CCTCAACCTGGCGGATGCG -3'		This study
rDNA-F	5'- GACCTTGCGCGATTGAATG-3'	75	Chen et al. 2011
rDNA-R	5'- CGGATCGTCGCCTTGGT-3'		Chen et al. 2011

Chen, C. H., Huang, C. C., Chung, T. C., Hu, R. M., Huang, Y. W., and Yang, T. C. (2011). Contribution of resistance-nodulation-division efflux pump operon *smeU1-V-W-U2-X* to multidrug resistance of *Stenotrophomonas maltophilia*. *Antimicrob. Agents Chemother.* 55, 5826-5833.
